# Exploring engagement with digital screens for collecting patient feedback in clinical waiting rooms: The role of touch and place

**DOI:** 10.1177/1363459319889097

**Published:** 2019-12-09

**Authors:** Bie Nio Ong, Caroline Sanders

**Affiliations:** NIHR School for Primary Care Research, University of Manchester, UK; NIHR School for Primary Care Research, NIHR Greater Manchester Patient Safety Translational Research Centre, Manchester, UK

**Keywords:** ethnography, health policy, technology in healthcare

## Abstract

Health service settings are increasingly installing digital devices to enable people to engage digitally with multiple processes, including automated ‘check-in’, as well as collecting feedback on experiences of care. In addition, policy is increasingly driving digital agendas to promote patient engagement with online services, management of health records and routine monitoring. While this tendency towards widespread digital diffusion has been viewed as a means of enabling greater empowerment of patients and improved responsiveness of services to ‘patient voice’, social scientists have provided critical insights on the use of digital technologies in practice. However, there remains limited understanding of the mechanisms and contexts for digital engagement. In particular, there is a need for further research on the sensory and spatial aspects of engagement that are integral to everyday use (or non-use) of technology in practice. This article reports new insights from detailed qualitative case studies utilising in-depth interviews with patients, carers and staff, in addition to ethnographic observations of different digital modalities and their usage in specific health care contexts. A sociomaterial approach and concepts of affective atmosphere and technogeography are drawn upon to analyse the role of touch and place in the collection of digital feedback in multiple waiting room settings for people with physical and mental health long-term conditions. The findings highlight how barriers to engagement varied by context such as particular concerns about privacy for those with mental health problems and physical and sensory barriers for those with physical impairments. The findings demonstrate how digital inequalities can play out in practice and have implications for the design and development of digital innovations and tackling inequalities that may be associated with implementation of new digital technologies in healthcare.

## Introduction

The English National Health Service (NHS) has adopted a routine approach to collecting patient feedback data for the purpose of improving quality and safety of care. Such feedback is commonly collected via the so-called Friends and Family Test (FFT) where patients are asked to indicate whether they would recommend a service to friends and family using a 5-point scale from ‘extremely likely’ to ‘extremely unlikely’. FFT data can be collected as a pen and paper exercise but are increasingly being administered through digital means in order to enable ‘real-time’ data collection. Digital feedback has been collected widely using a text messaging service, but increasingly health service settings are installing devices to enable people to engage digitally with multiple processes, including automated ‘check-in’, as well as collecting feedback. In addition, policy is increasingly driving digital agendas to promote patient engagement with online services, management of health records and routine monitoring. While the digitisation of care systems and services requires different types of engagement for patients and staff, all require some level of interaction with associated technological tools, whether that be for collecting data, organising or receiving care, or reporting on experience of care. This tendency towards widespread digital diffusion has been viewed as a means of enabling greater empowerment of patients and improved responsiveness of services to ‘patient voice’; however, social scientists have offered critical reflections on the practices of collecting and using feedback (e.g. Lupton, 2014a; Mazanderani et al., 2012; Ziewitz, 2017) as well as potential for digital inequalities according to variables such as age, material status, gender, ethnicity and disability (e.g. Barbosa Neves et al., 2018; Lupton, 2014b; Ragnedda and Muschert, 2018; Robinson et al., 2015). However, much research on digital inequalities has been based on quantitative analysis of patterns of use within large data sets and there have been calls to avoid simplistic notions of a digital divide (Halford and Savage, 2010). A more nuanced understanding of the mechanisms and contexts for digital engagement is required, and one way forward is to carry out detailed case studies of different digital modalities and their usage in specific health care contexts.

The rapidly growing literature on digital technologies in health offers a number of concepts that are particularly relevant in framing our thinking about digitally collected patient feedback. First, Lupton (2017) argues that the concept of affective atmosphere can elucidate the sociocultural dimensions of digital health technologies. She defines affective atmosphere as referring to ‘the feelings that are generated by the interactions and movements of human and nonhuman actors in specific spaces and places’ (p. 1). Similar to others adopting a sociomaterial approach, close attention is paid to the role of non-human actors (e.g. objects, spaces, places) when researching healthcare technologies in practice (Buse et al., 2018; Mol et al., 2010; Oudshoorn, 2012). Such approaches allow for a wider perspective in that the emphasis shifts from what people say they do when interacting with digital tools to how they act or feel. Thus, the focus becomes the embodied human practice within a sociomaterial context.

This study focuses on the introduction of new digital patient feedback tools (inviting feedback on care received via ipad kiosks) within waiting room environments across multiple healthcare settings. The waiting room can be defined as the place where individuals experience an ‘in between’ state transitioning from citizen to patient and where they are generally in a passive state of waiting for their consultation. In contrast, the kiosk requires an active engagement and the individual becomes the patient as consumer providing feedback on the service they have received. In this context, the interaction between the human actors such as patients, reception staff or clinicians on one hand and non-human actors such as the digital screens or spaces where these are situated on the other hand is key to understanding how and why these modalities are adopted (or not). Lupton (2017) has extended analysis of material interactions with digital tools to the concept of affective atmosphere helping to surface often ‘barely conscious feelings’ that can motivate people to engage with digital feedback tools and how these feelings are influenced by collective and relational sense-making. Oudshoorn (2012) introduces the notion of technogeography to emphasise how the use and meaning of technical devices are dependent on place, such that the same technological device can do and mean different things in different places. In this way, places shape how technological devices are used, or not, and interpreted. Conversely, technologies also contribute to shaping the meaning and practices of the spaces in which they are employed, and how people and objects interact in those spaces. While collecting feedback via a digital kiosk is quite distinct from engaging patients in the delivery of care via the technology, the concept of technogeography has relevance for understanding the use and meaning of devices in relation to place and relationships between patients and healthcare staff. This is because of key concerns about trust and privacy and threats to patient identity where recipients of care might be labelled as ‘difficult’ or ‘demanding’ if they give poor feedback. Such issues have potential to transform the waiting room space as an extension of the clinic space where power relations facilitate different experiences of waiting (Bates, 2019) and where waiting is ‘performed’ (Brown et al., 2019).

A second area of relevance is research on touch, and this has been of interest for many disciplines including anthropology, experimental psychology and neuroscience. Jewitt and Leder Mackley (2019) state that touch is central to human existence and that a social analysis is pivotal to a better understanding of the impact that digital touch technologies have on society and individuals. They argue that the sensory experience is foregrounded in the development of digital touch devices and that the social sciences need to respond with appropriate theoretical and methodological approaches to analyse these new sensory communications. In doing so, they discuss the contributions of multimodality and sensory ethnography to researching touch. ‘Multimodality is concerned with the agency of people and the politics of change (authors emphasis), while sensory ethnography foregrounds notions of emergence, imagination and ongoingness’ (p. 105). The latter involves emphatic understanding of people’s actions and their ways of knowing, being and doing. In their paper they advance a dialogue between the two approaches and conclude that it aids the articulation of ‘the relationship between the sensory and modal aspects of touch communication’; allows us ‘to connect different analytical levels of experiences of touch across individual sensory perception to socially-culturally shaped modes and norms of touch’ (p. 106); and stimulates a more reflexive approach to making sense of digital touch in varying contexts. Finally, Paterson (2009) argues that touch operates as a broad sensory modality and that it is always mediated through psycho-physiological features. By employing digital technologies touch becomes remediated.

Third, Richardson (2010) argues that the human–technology relationship is a body–tool relationship, and openness to the digital environment allows individuals to incorporate new digital tools and technologies into their physical world. This world is shaped by sociocultural, environmental and historical factors and also by person-specific psychological and physical dimensions thus leading to variable individual embodied responses to digital devices. A further extension of this argument is represented in the work by Pink and colleagues (2016) who draw attention to the haptic sensations that are encountered by the hand when touching digital devices and to how knowledge is gained through the hand. They build on the insights of earlier research (Ingold, 2013; Verhoeff, 2012) that focuses on the ‘haptic engagement’ of the hand with digital touch screens and its capacity to both know and tell. This literature conceptualises the hand as an extension of the brain and by being actively engaged with its environment it combines sensory perception (touch) and embodied knowledge with affective and emotional ways of feeling. This resonates with the work of Pols and Moser (2009) who describe the values that are brought into play when analysing relations between technologies and their users and how emotions are evident. They question the opposition between ‘cold’ technology and ‘warm’ care and show how different affective and social relations between people and technologies emerge in different contexts.

The focus of our article is on the physical engagement with a kiosk consisting of a digital touch screen to collect patient feedback data. We are particularly interested in the haptic interaction of users within the contexts of primary care, acute hospital (rheumatology outpatient) service and mental health services. We begin with outlining the study, design and methods, followed by the presentation of our findings from both observations and interviews. The discussion will be based on the literature cited above that provides a set of concepts to aid the understanding of the social processes of digital engagement as shaped by agency and experience. The analysis of spatial and embodied aspects of interactions with the digital kiosks also draws to the fore implications for digital inequalities when introducing new technologies requiring input from those who already face disadvantage and social exclusion due to physical impairment or mental health problems.

## The DEPEND study

This article reports on an evaluation of a digital patient feedback system within four health service organisations (Acute Trust, Mental Health Trust and two General Practices). This component was nested within a larger mixed methods study entitled ‘*D*eveloping and *E*nhancing the Usefulness of *P*atient *E*xperience and *N*arrative Data (DEPEND: HS&DR ref – 14/156/16). The DEPEND study aimed to understand how to improve the credibility, usefulness and relevance of patient experience data in services for people with long-term conditions using digital data capture and improved analysis of narrative data. The DEPEND study was designed as a mixed methods study with four parts: (1) the collection and usefulness of qualitative research data to explore perspectives of patients and carers and staff on current experience, and what needs to improve; (2) the analysis and presentation of patient feedback data by utilising computer science text analytics methods to develop routine, semi-automated analysis of free-text feedback comments; (3) the co-design of a toolkit of new digital and non-digital tools to support the capture, analysis and use of patient feedback and (4) the implementation of the toolkit and a process evaluation using Normalisation Process Theory (NPT) to assess the use of the new tools.

The tools designed were a survey to complete digitally via tablet device (a kiosk) in waiting areas or alternatives (pen and paper/online version); guidance and information for patients, carers and staff; text mining programmes; reporting templates and a process for eliciting and recording verbal feedback in community mental health services (reported elsewhere).

## Participants and data collection

There were four study sites: a rheumatology outpatient department in a large acute Trust (Site A), a community mental health team and outpatient clinic in a Mental Health Trust (Site B), and two general practices within the same locality (Sites C1 and C2). Each setting had varied existing practices for collecting patient feedback, with predominant reliance upon pen- and paper-based surveys with low levels of participation, and challenges for collecting and processing responses. Of note, Sites A and C2 collected digital feedback via text message, and Site A occasionally collected digital feedback from selected inpatients and outpatients using handheld digital devices.

Participants in the implementation and evaluation component of DEPEND included staff, patients and carers, and qualitative data were generated via large focus groups, individual interviews and observation methods. Our rationale for using all these methods was to develop a focus on in-depth personal narratives via qualitative interviews and a multi-dimensional perspective via interactions within the focus groups; as well as observing practices and patients/carers in the use of the kiosk. The samples reflect maximum variation (Patton, 1990) by including a balance of patient and carer participants in terms of gender, age and ethnicity, as far as possible. For staff sampling, we ensured diversity in terms of roles and experience. The study design allowed for in-depth investigation and enabled triangulation of the data and emerging key themes.

Following the introduction of the new tools and guidance, individual interviews were conducted by one of the two project researchers and focus groups were facilitated by two to three members of the DEPEND team (main topics and prompts covered within focus groups/ interviews are summarised in Table 1). Interviews and focus groups were carried out with 51 staff participants, 24 patients and 8 carers (see Table 2).

**Table 1.** Summary of topics and suggested prompts for focus groups and interviews with staff and patients.

Topics and prompts for interviews/focus groups with staff	Topics and prompts for interviews and focus groups with patients
*Making sense of why the toolkit was being implemented* • Did you see the effort being put into overhauling the way that patient experience data were being collected, processed and presented as being worthwhile? ○ Were the previous ways of doing this work effective? ○ Do you see value in collecting patient experience data? ○ Is patient experience data generally put to good use? • Did you feel that the training had given you an adequate understanding of the new toolkit when it was implemented? • How different do you see the new toolkit from the previous system that was in place? ○ Was it seen as an improvement, no different or creating more problems? *Examining the extent to which staff bought in to the use of the toolkit* • Do you think that the responsibilities for implementing the toolkit have been shared equally among your colleagues, or was there a disproportionate burden taken by any one person or group of people? ○ Prior to implementing the toolkit, did you have concerns that using it would increase your workload or responsibilities? (Note the different implications for managers (leads for patient experience) and for Unit staff) ○ How has implementation affected your workload and way of working? ○ How appropriate is it that you or the group of which you are a part take on any additional responsibility? • How well have staff (IT, management and healthcare professionals) worked together to implement the toolkit? • Were there any factors occurring at the time within your work environment that you feel affected your involvement with the toolkit’s implementation? For example, dept. restructuring, staff shortages, reassignment of responsibilities, job uncertainty, etc	*Experience of the newly implemented resources* • Ask participants to describe their use of the newly implemented resources to collect patient experience data ○ What were your initial impressions of the technology that was being used to collect the data? ○ To what extent did you feel that the input that was given in the earlier phases of the study was incorporated into the new means of collecting data? • Was the input given worthwhile? ○ How comfortable were you using the new system? • Were the instructions as to how to input your responses adequate? • To what extent was it self-explanatory what you needed to do to respond? ○ What did you like about it? What didn’t you like? • How relevant were the questions that were asked? • Were the response options appropriate? • What did you think of how question items were displayed? ○ What did you think about the length of time that it took to complete the questions that were asked? • Did you experience any problems providing details of your service experience? ○ Were there any technical difficulties? ○ If you did experience any difficulties, how did you resolve them?
Operationalising use of the new toolkit • How has the toolkit been used in practice? • Since the implementation of the toolkit, have you noticed any change in the level of interest/encouragement/support shown to patients to provide experience data? • Do you now feel any better equipped to take advantage of patient experience data as a result of how it has been analysed and is now presented? • Have you noticed any changes taking place as a result of the data collected? *Appraisal of the toolkit* • Have you been aware of any attempts to review or monitor the operation of the toolkit within your Unit? ○ How was this done? • Has there been any need to modify how the toolkit has been employed as a result of the experience gained from its use? • What benefits/deficits over the previous way of working did you identify regarding: ○ The new way of collecting patient experience data ○ How data were presented? • Does the new system for collecting data appear to have affected patient engagement?	• How does this system of collecting data rate against the one that had previously been in place? ○ To what extent have the changes been sympathetic to the difficulties that you personally experience as a service user? ○ Can you make any suggestions as to how the experience of reporting patient satisfaction data would be more satisfying? Such as in terms of • The technology used • How the questions were presented • On the basis of this experience, how encouraged would you be to take part in giving feedback again in the future? *Final thoughts* • Is there anything that the interviewee would like to add to the discussion?

**Table 2.** Interview participants by sites: implementation and evaluation of a digital patient feedback system.

Participants	Sites				Total
	A	B	C1	C2	
Staff focus groups	5	19	8	8	40
Staff interviews	0	7	2	2	11
Total staff	5	26	10	10	51
Patient focus groups	0	13	0	4	17
Patient interviews	6	0	0	1	7
Total patients	6	13	0	5	24
Carer focus groups	0	2	0	1	3
Carer interviews	1	4	0	0	5
Total carers	1	6	0	1	8
Grand total	12	45	10	16	83

Focused observation (42 sessions) was used as this type of data collection underlines the importance of organisational and contextual elements that shape human behaviour (Morgan et al., 2017). Moreover, as qualitative researchers we were aware that what research participants say and do needed to be interpreted alongside the material and sensorial settings in which they operate (Hurdley and Dicks, 2011). Thus, the observations helped to understand how the new tools were utilised, and how resulting data were used by staff. Full NHS Research Ethics Approval was obtained for the DEPEND study (Black Country NRES committee in West Midlands ref: 16/WM/0243), and all participants gave written consent.

## Data analysis

All interviews and focus groups were transcribed, collated and analysed thematically drawing on a grounded theory approach (Strauss and Corbin, 1998) and using NVivo11 qualitative analysis software. Detailed observational notes from weekly visits to the site were written up by the two project researchers and circulated among the research team to aid the analysis. Two members of the Patient Participatory Group (PPG) attached to Site C2 were also able to contribute reflections based on their experiences of helping to promote the kiosk. Open coding was conducted by two researchers to provide an initial framework and corresponding descriptive accounts for the multiple sets of data producing distinct accounts for each participant group (patients, carers and staff) and for each site. These were used as a basis for discussion at regular meetings with the research team and members of the Patient and Public Involvement Advisory Group where links and distinctions across the multiple groups of participants and case study sites were explored.

## Findings

### Physical engagement within different environments

**Figure 1.**
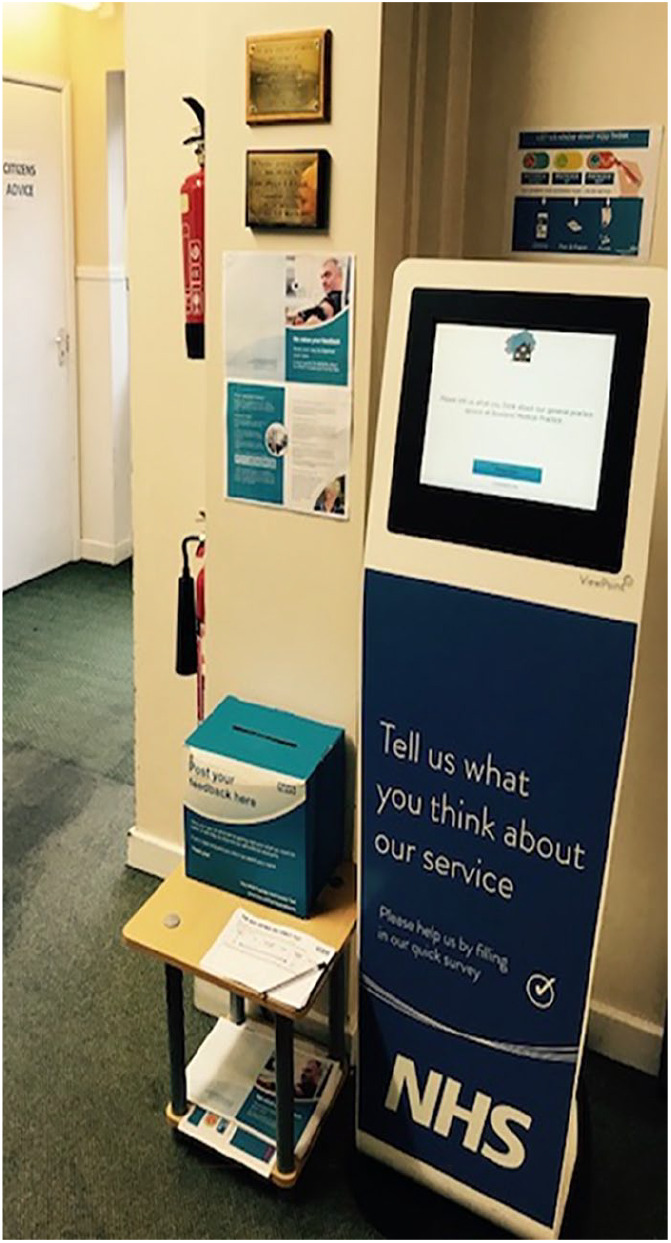
Photograph to illustrate the space and positioning of the data entry screen in Site C2.

**Figure 2.**
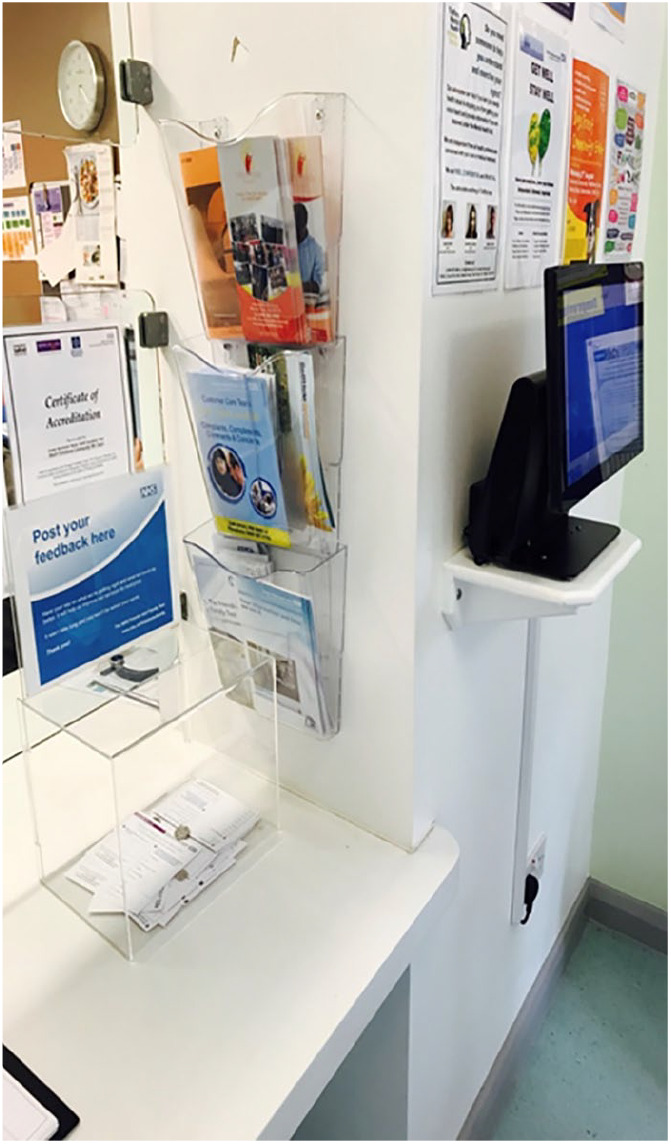
Photograph to illustrate space and positioning of the data entry screen in Site B.

In all four sites, the kiosk was situated in the waiting areas. In Sites A, C1 and C2, the kiosks were stand alone units (see Figure 1), while in Site B the touch screen was placed on a shelf close to the reception desk (see Figure 2).

In Site A, the kiosk was moved to a more prominent place a few weeks after the start of the fieldwork following a discussion between the outpatient department manager, a researcher and volunteer and the explanation was contained in the following observation:

I think the Kiosk has not been used much since [installation]. I have asked four doctors about this and none of them have seen anyone using it . . . I have [also] spoken to some support staff (n = 4). Again, none of them have seen anyone using it. Some of them haven’t even seen the Kiosk and came with me to see it. This is partly because the Kiosk was placed in a hidden spot, as you know . . . I have been trying . . . to find out if it is possible to bring it in a place where it would be more visible . . . [staff] helped me to move it in front of the reception desk. After moving the Kiosk in front of the reception desk, there were 9 participants who gave feedback [in 1 hour and 15 mins]; whereas in the first hour there were none. (Observation note, Site A)

**Figure 3.**
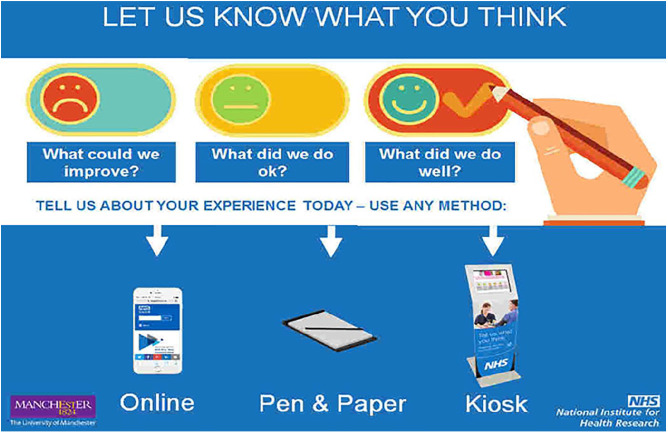
Poster situated on the wall immediately above the kiosk inviting patients and carers to provide feedback.

The co-location of the kiosk with the enlarged explanatory poster (Figure 3) was also designed to make the kiosk more prominent and inviting.

From the patients’ perspective, the primary function of the waiting room is exactly that waiting and preparing for a clinical encounter. The time between arrival and seeing their clinician is focused on being ready when called to attend. Reading information on posters or leaflets may occupy their attention but is bounded by the cut-off point of their consultation. Thus, requesting patients in the waiting room to concentrate on giving feedback is a challenge because the window of opportunity is unpredictable. Asking people to engage with a digital device further exacerbates this challenge:

[. . .] However she had noticed the kiosk before now but did not use it. I asked why this was and she said because she’s always in a rush and she had time now to use it as her care coordinator was running late. (Observation note, Site B, 10 August 2017)

Conversely, other patients felt that the digital modality was attractive because it was quick and giving feedback could be done within the waiting time frame available:

Well, I think the idea of the digital . . . yeah, pressing the box is quite a good one, because it’s relatively quick, it’s straightforward, it’s easy to deal with. (ID 115, patient interview, Site A)

Patients’ perceptions of whether they can fit in giving their feedback is shaped both by their assessment of the time needed to respond and whether this can be achieved within the period they are waiting to be called or following their appointment. This also seemed to have a bearing on the nature of responses given, as a key concern of service providers was to enable data that is sufficiently ‘rich’ to be collected in order to capture some detailed context regarding patients’ experiences in addition to the tick box response to the FFT. However, the observations indicated that comments being entered were extremely limited, for example, ‘very good’.

The waiting room as a shared public space poses a potential conundrum: filling in a questionnaire about your experience of care is generally considered a private matter, but doing that in view of others may be uncomfortable. For example, one patient at C1 declined to use the kiosk as she did not want to appear as a fault finder. The woman said,

‘the kiosk is ok but standing in front of the machine is like a proof of dissatisfaction towards the service. When you stand here, everybody will see that you are giving feedback. You don’t want to identify yourself as a faultfinder, do you?’. I said: ‘but people can give positive feedback’. She said: ‘well, everyone knows that no one gives feedback to praise any one. Like me, I am giving feedback because there was something wrong there’ (she pointed to the doctor’s door). (Observation note, C1)

The location of the touchscreen on the reception desk in Site B made this even more poignant as health care staff sat often close to the stand next to the reception desk and highlighting tensions between the public and private, for example,

The [clinic nurse] said she wondered if it was the best site to collect digital feedback data using this device as the small reception area has privacy issues and people may feel pressure to use it and that’s why not many people have to date. (Observation note, Site B)

A number of service users were observed using the kiosk, but overall participation was low, and one person commented on the location to the researcher:

She commented that she would be more likely to use it if the kiosk was located near to the door on the way out. She also thought that younger people would be more likely to use it than older people, as a ‘form of distraction, maybe . . .’ She thought that it could be advertised more prominently than it was as she did not associate the kiosk with the A4 poster above the kiosk on the wall (behind a glass cabinet). (Site B, observation note)

Thus, location of the kiosk was an issue as a balance had to be found between it being visible and accessible while affording sufficient privacy for individual users. Furthermore, clarity as to the purpose of the kiosk and the audience it targeted proved to be ambiguous for some people:

because it was my sister’s appointment, she is a patient, so I didn’t know I was allowed to touch that . . . I think people just walk in and presume that it’s just for patients to let their doctors know that they are there or something like that. (Site B Outpatients, ID 263, interview)

In all sites, there was greater engagement with putting in data when the researchers were present and entering into discussions with patients and carers. In Site C2, this was also boosted by support from volunteers who developed a rota to offer peer support within the practice waiting area. They talked about their own observations on the way people engaged with the kiosk, as well as support needs:

[The volunteer] noticed that younger people seemed to be a lot quicker at typing their experiences, whereas the middle aged took their time, and the elderly wanted to try the kiosk with the help from a volunteer. (Observation note, C2)

In all other sites, provisional discussions had taken place and support was given regarding the involvement of volunteers to act as peer supporters for using the kiosks. However, this system was not routinely adopted due to competing priorities for the small groups of volunteers.

The perspectives of staff on their role in relation to support for collection of feedback also varied between the different sites. For example, several of the doctors in Site A were concerned that it was ethically problematic for them to inform patients that they are able to give feedback using the kiosk as summarised in one observation note that

if the doctors tell the patients to give feedback, the patients will think that they are supposed to give feedback only about that particular doctor. And that can be misleading and provide bias information. Therefore they have decided not to mention it to the patients. He also told me that they have discussed this issue during their Governing meeting this week, and they think that instead of doctors the other support staff should mention to the patients to give feedback. They can tell them when they are collecting blood, measuring blood pressure or providing other samples. (Observation note 4, Site A)

Lupton’s (2017) discussion of affective atmosphere draws attention to the importance of the human–non-human interactions within specific environments. The waiting room is socially defined as a public environment with the function to provide space for patients to wait and prepare for their clinical encounter. Placing a digital kiosk within this space demands patients to recalibrate their expectation of appropriate performance which has led to responses ranging from ignoring the kiosk, feeling confused about whether and how to approach it through to engagement. Furthermore, as the above examples illustrate the data demonstrate various adjustments and changes were made throughout the study period to navigate spatial challenges for engagement, but where this threatened to disrupt established norms and ‘ethics’ for professional–patient relationships, resistance was evident.

## Touching the screen and inputting data

**Figure 4.**
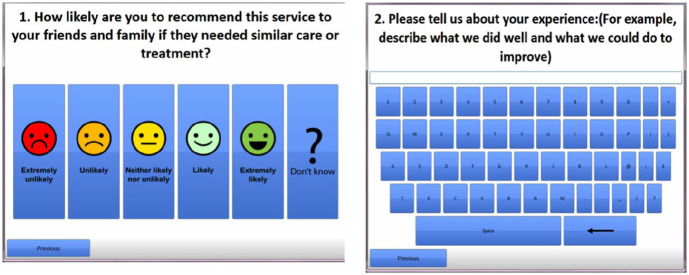
Interface of FFT question and question asking for free text comments regarding experiences of the service.

The touchscreen questionnaire displays the FFT in six interfaces, one of which allows for open-ended comments (see Figure 4).

We focus our discussion on the fourth interface (Figure 4) where people can write down their own experiences. The use of a touchscreen posed issues for a number of people who had no previous experience with it. Comparisons were made with traditional keyboards such as by this woman who told the researcher:

[. . .] This touch screen is too flat for me, not good for me. I need a keyboard to type, I cannot type in it. (Observation note, Site C2, 7 September 2017)

More specific problems were mentioned by a number of people, for example:

[. . .] delete button is not easy. If you have to delete one thing you, which is in the middle of the sentence, you have to delete everything. On the other hand, when we write in the text message the cursor can easily be moveable to the word that you want to delete. But that is not the case here. (Observation note, Site C2, 7 September 2017)

This woman compared her experience of deleting letters on her mobile phone with the touchscreen and expressed concern that her knowledge appeared not to be transferable. Furthermore, while she was familiar with working on computers, the difficulty with the delete button caused her to ‘feel nervous’ as she was worried that everything she had written ‘would vanish’.

Several people mentioned the problem of the need to apply pressure on the letters. Comparisons were made with devices that individuals used themselves, such as this woman who commented that the keyboard ‘should be more like an ipad screen as there is a pressure burden behind it at the moment’ (Observation note, Site B, 5 September 2017).

For patients who had hand impairments, this was even more of an issue. One woman who was wearing special gloves for her arthritis said,

It hurts, I cannot type with this. (Observation note, Site A, 4 September 2017)

Similarly, at the same site, another woman asked her husband to type in her place as she also wore gloves. Other people tried to adapt their engagement such as the following two people: ‘Two of the patients were slowly typing with their left hands as both of them had bandages on their right hand for arthritis problem’ (Observation note, Site A, 31 July 2017).

It appeared that some patients had built up knowledge through their hands (Verhoeff, 2012) from past experience with digital devices and the way in which they could adapt their engagement despite physical impairments. Others knew that they could not operate the touchscreen because it caused pain and discomfort and their hands could ‘tell’ (Ingold, 2013) that engagement would not be possible.

Impairments in other parts of the body impacted on the way people’s hands could perform, and two cases exemplified this situation. First, wheelchair users could not reach the touchscreen as it was placed too high:

Let’s look at the other side of the coin. People in wheelchairs, are they going to find it as convenient as an able bodied person that can stand up and operate a screen? (ID225, patient, Site A)

The second example was as follows: ‘ One of them was using crutches (I hesitantly requested her to take part) and she managed to stand holding one and typed with the other hand’ (Observation note, Site A, 4 July 2017).

Richardson (2010) notes that different types of digital screens demand variable embodied orientations involving an ‘immersive investment’ of the senses, including the hand. Individuals with a physical impairment are compromised in their ability to achieve this investment and are either excluded (like the wheelchair user) or find it more difficult to complete the task (the person with two crutches).

The close interplay between the hand and the eyes has been highlighted by several authors (Pink et al., 2016; Richardson, 2010) and is demonstrated in our study. Visual issues emerged with many individuals, exemplified as follows:

The room (typing box) for typing was not visible. It needs a big space. You cannot see when you type. When the writing is going on you cannot see. I have seen them eventually. It’s not obviously visible. It took me some time to find where the typing going? (Observation note of verbatim comment, Site C2, 7 September 2017)

Another woman also could not read the kiosk properly [due to visual impairment]. She started but then she said, ‘I cannot see the write up but I can see the pictures’. Her daughter helps her to fill it out at the end. (Observation note, Site A, 26 July 2017)

In the same way as other physical impairments, not being able to coordinate the eyes with the hand made it difficult for people to use the touchscreen: in the first case, it slowed the person down, and in the second case, she had to abandon her effort, but could get her daughter to replace her.

A final concern that was raised related to lay beliefs about transmission of germs through the hand. One participant was concerned about the way the touchscreen was generally kept clean and suggested that ‘hygiene lotion could be placed next to the kiosk’ (Observation note, Site C1, 15 August 2017).

Two women highlighted both sides of the contagion fear:

[. . .] woman disagreed to give feedback in kiosk said, ‘sorry I am not going to give my feedback today via this machine. Don’t get me wrong, I have seen someone with flu touching it, don’t get me wrong. I don’t want to do it today. I don’t want to get me flu again. Already I have been going through a lot’. She then promised that she would do some other day [. . .]. (Observation note, Site C2, 29 September 2017)

And:

A woman said, I am having flu and I don’t want to touch this screen to spread it. (Observation note, site, Site C2, 17 October 2017)

The notion of contagion has been prevalent in anthropological literature and different models of contagion have been formulated (Nemeroff and Rozin, 1994). One of those models is microbial contagion based on the scientific germ theory. This model is often incorporated within the lay models of contagion and in the above observations of the use of the touchscreen this became apparent. The appropriation by lay people of biomedical knowledge has been recognised as a way of making sense of illness and its origins (McClean and Shaw (2005), and thus, the above reasoning is consistent with this broader tendency. It also resonates with recent sociological research where interaction and spatiotemporal organisation of care within clinical waiting areas reflects their designation as cross-infection ‘hot spots’ (Brown et al., 2019).

## Continued engagement or disengagement

While we have mainly reported on the difficulties people encountered when using the kiosk, a large number of individuals successfully completed the questionnaire, particularly those who were already familiar with touchscreens:

I think the fact that you’ve got . . . I mean, like the idea, for example, the touchscreen . . . I mean, I’m just thinking again of like the GP surgery where you automatically go in and you book in . . . I think that’s quite a good way of doing it, because it’s quite visual, it’s automatically recorded. (Patient interview, ID 115, Site A)

Another reason given for using the touchscreen was the perceived quickness of answering the questions compared to pen and paper and fitting in with the limited timescale available:

I think the screen is an attraction because it’s standing there, and people might think it’s quicker than hanging about if they’ve not got a pen, if they make a mistake on the form, whereas if they make a mistake, they can go . . . if they know how to go back on it, to change it. (Patient interview, ID 134, Site C1)

The idea of fast responses was taken sometimes quite literally as the example of this young woman demonstrates:

I asked her why she was so quick at typing and she said that she would be more likely to write a longer comment on the postcard. I asked why this was and she said that the digital device was so quick to use and its purpose is to collect ‘real time feedback’ so this prompted her to be quick with her typing! (Observation note, Site B, 12 October 2017)

In general, the people who perceived the touchscreen to be easy and fast were motivated to approach the kiosk and fill in the questionnaire. Previous experience of digital touch and knowledge by the hand facilitated engagement. Actual completion could not always be achieved if they were called in for their consultation. This gave rise to problems which related to the technological shortcomings:

One patient was willing to give feedback after getting consultation from the doctor while she was still waiting for blood test to be done. When she was typing on the Kiosk, the nurse called her for blood collection. She then left the kiosk immediately and gone for blood test. The page that she was typing disappeared when she returned from the blood room. She had to type everything again. (Observation note, Site A, 31 July 2017)

In this case, the patient was resigned to repeating her efforts, but others abandoned the exercise.

A similar technical problem was that the machine operated with an automated timer:

When a man was filling out the kiosk survey and he was typing in question 2, he stopped typing for a few minutes as he was trying to find out the doctor’s name from his prescription. By the time he found the name and ready to type it, the kiosk screen went back to the first page automatically. He then started giving information again from the first page. At the end, he told me this and said, ‘it was too quick’! (Observation note, Site A, 18 July 2017)

While this man was willing to start from the beginning again it was clearly frustrating and could have led to disengagement. The same happened at a different site and the patient offered a solution:

W [volunteer] helped the man through the digital survey. However, when he got to the free text comments question the receptionist called him over and he left the kiosk. He came back and had to retype all his answers again, he suggested the use of a pause button would be useful in this situation. (Observation note, Site C2, 11 August 2017)

The above technical problems could lead to people aborting their attempts, or not returning after their consultation. A further issue related to the concept of time, but different than the one mentioned earlier: because the touchscreen was perceived to be a faster method for giving feedback it created pressure on thinking, such as in the following example:

She said: ‘the kiosk is not a good idea’ as she has to think there and then when standing in front for the computer (kiosk**).** She cannot think so quickly these days. She needs some time so she will do it from home. (Observation note, Site A, 6 September 2017)

This form of non-engagement appeared more prevalent with people who were not familiar with computers, who were older or who suffered with a mental health problem. They would like to offer their views but the digital modality did not suit them, and if it was the only option this could lead to inequality.

The experiences of completing of using the touchscreen appeared to range from highly acceptable to disengagement. Those people who were digitally literate appreciated the ease and speed of the kiosk; others who for different reasons did not have the same knowledge and familiarity found it difficult and either discontinued their responses or did not start at all.

## Discussion

The DEPEND study focused on the acceptability and use of digital patient feedback, and in this article, we discussed the specific theme of the psycho-physiological features of digital engagement within specific contexts. The underlying rationale for this can be traced back to the work of Merleau-Ponty (2003), who saw the lived body perspective as allowing us to understand the body not simply as a thing-in-the-world but as a medium in our sense-making practices because, unlike inanimate objects, it is intentional and directed towards an experiencing world. Authors who have a particular interest in digital touch have taken this further and argued that touch screens transform the embodied experience of sociality and material culture (Jewitt and Leder Mackley, 2019). Moreover, in order to understand where touch happens and how it is experienced and made sense of, the contexts within which touch takes place needs to be taken into account (Ingold, 2013; Lupton, 2017). Health geographers have broadened their examination of the meaning of place to include digital spaces such as social media platforms and developed ecological frameworks to explain the complex and multi-layered relationship between the technological modalities and well-being (Shankardass et al., 2019). Similarly, researchers exploring engagement with digital technologies have provided rich ethnographic insights into the role of objects, spaces and places, as well as human interactions, whereby affective and social relations are shaped in specific contexts (Lupton, 2017; Pols and Moser, 2009).

In the DEPEND study, the physical spaces derive their meaning from the health care system and those working within it as well as from the patients and carers. The nature of the waiting room as a place reflecting the transition from passive and ‘waiting’ citizens to patient as active consumers can be managed by most people if it is bracketed off within time and space, but the main problem highlighted in our study was the uncertainty about the time available to complete the questionnaire. People familiar with digital tools were quickly able to achieve completion, but many others were not, and comments provided were often limited to very few words. This is very different to the type of feedback sometimes seen via online forums such as ‘Care Opinion’^1^ where much longer accounts are quite commonly provided. For the latter, people can enter the data anywhere via the Internet and would tend to do this in private space of their own homes. This further draws attention to the combined material and sensorial influences on how digital data are generated indicated in Lupton’s notion of ‘affective atmosphere’ and Oudshoorn’s notion of ‘technogeography’. In addition, the interaction between patient/carers and health care staff or volunteers with the aim to support them in using the kiosk became a vital element to successful engagement. However, there were also perceived conflicts and uncertainties regarding the involvement of staff and this is important because new technologies can only make sense within the social relations in which they need to function (Harland and Melby, 2015).

The data draw attention to different sensitivities reflected in actions around engagement or lack of engagement that seem entangled in the affective atmosphere and technogeography of specific contexts. For example, there were more specific concerns about ‘privacy’ and the sensitive nature of feedback for people using mental health services. This seemed to be a contributing factor in the particularly low levels of engagement and represented in views about the need to ensure options for alternative modes of feedback (verbal, interactional, informal). This resonates with the work of Pols and Moser (2009) on how values and emotions are reflected in interactions around care technologies.

When problems were encountered, the importance of materiality, embodiment and digital touch became apparent. The examples of people who were wheelchair users or walked with crutches demonstrated that their use of the kiosk was compromised as they could not stand in the right position to operate the touchscreen. Richardson (2010) has pointed out that the location of the touchscreen and the body within the built environment of the waiting room, the functionality of the technology and the spatial arrangement all play a role in influencing engagement. If alignment is missing, either individuals have to adapt, such as the person with the crutches propping themselves up and typing slowly, or not participate and digital exclusion ensues.

The examples of people thinking that the touchscreen is difficult to operate because of problems with their hands such as finding the pressure required too high highlight that touch is key to using digital technology. Jewitt and Leder Mackley (2019) argue that touchscreens transform the bodily experience of sociality and that the sensory experience of the community of patients and carers helps to redefine their thinking about feedback. In the case of DEPEND offering the kiosk as the sole mechanism can be perceived as not sufficiently valuing patient feedback if people are not able to use alternative methods such as conventional keyboards, telephone, or verbal feedback. The call by several participants to place pen and paper alongside the touchscreen attests to the need to provide other options.

The close immersive investment of the eye and hands that touchscreens demand of its users was pointed out by Richardson (2010). It also chimes with Ingold’s (2013) conception of the hand as an extension of the brain, and by being active in the world ‘knowing through the hand’ accumulates. This was borne out in our study where patients talked about their inability to see the letters clearly were worried about texts ‘disappearing’ or not fitting in the allocated space. Attending to these concerns was essential for engagement to be achieved and maintained and staving off anxiety or disengagement. Often, support by volunteers helped in this respect and individuals were able to complete the questionnaire. By doing so, they increased their confidence and the capacity of their hands as ‘repositories of memory’ (Ingold, 2013).

## Conclusion

The findings reported in this article have provided new insights on the sensory and spatial aspects of touch and place associated with digital engagement that demonstrates the significance of affective and social relations between people and technologies that emerge in different contexts when providing feedback on care received. While such issues have been given limited attention in previous research on remote care, our analysis demonstrates how engagement (or not) of new digital technologies can reflect an extension and transformation of the spaces of clinical care, and in this case how the passive space of the waiting area takes on new expectations requiring active input from patients with new implications for trust, privacy and patient–professional relationships. The analysis highlights how barriers to engagement varied by context such as concerns about privacy for those with mental health problems, and the physical and sensory barriers for those with physical impairments. Previous research on digital engagement has often focused on quantitative patterns of engagement with related qualitative research often focusing on perspectives of end users. However, this article demonstrates the importance of embodied interactions with digital technologies and the material contexts in which they are delivered. This analysis demonstrates how digital inequalities play out when patients who already face disadvantage and social exclusion associated with long-term health problems are invited to engage with new technologies in everyday care settings. In the context of collection and use of patient feedback focused on here, this means that the views and experiences of patients likely to have more severe problems will also be excluded from appraisals of the quality and safety of service delivery and associated plans for improvements. Recent quantitative analyses have provided some insights into patterning of digital engagement and corresponding digital divides, such as higher rates of non-use of the Internet among disabled people (Office of National Statistics (ONS), 2019). However, the current research serves as an exemplar towards understanding the nuances of digital inequalities in the health care domain at a time of increasing pressures to digitise health services that place new expectations on patients . The findings have implications for co-design and development of digital innovations and associated interventions to support digital engagement and towards tackling digital inequalities that may be associated with implementation of new digital technologies in healthcare.

## Notes

1.
https://www.careopinion.org.uk/
